# Long-term mortality and predictive score performance in Brazilian atherosclerotic renovascular disease patients

**DOI:** 10.1590/2175-8239-JBN-2024-0181en

**Published:** 2025-10-10

**Authors:** Julia Gheller Salome, Lucas Peres Moraes, Rodrigo Hagemann, Vanessa dos Santos Silva, Fabio Cardoso Carvalho, Roberto Jorge da Silva Franco, Diana Vassallo, Luis Cuadrado Martin, Philip A. Kalra, Pasqual Barretti

**Affiliations:** 1 Universidade Estadual Paulista, Faculdade de Medicina de Botucatu, Departamento de Clínica Médica, Serviço de Nefrologia, Botucatu, SP, Brazil.; 2 Universidade Federal do Paraná, Departamento de Clínica Médica, Serviço de Nefrologia, Curitiba, SP, Brazil.; 3 Mater Dei Hospital, Department of Medicine, Msida, Malta.; 4 University of Manchester, Salford Royal Hospital, Salford, United Kingdom.

**Keywords:** Atherosclerosis, Renal Artery Stenosis, Chronic Kidney Disease, Hypertension, Mortality

## Abstract

**Introduction:**

Atherosclerotic renovascular disease (ARVD) can cause renal artery stenosis, hypertension, and chronic kidney disease. As revascularization procedure for ARVD is controversial, a risk score was developed to predict mortality in affected patients, which requires validation in different populations. The original risk score did not include statin use; therefore, the aim of this study was to evaluate the accuracy of the risk score in ARVD patients according to statins intake.

**Methods:**

Longitudinal retrospective study involving 136 patients with angiographic diagnosis of RAS > 60% from January 1996 to October 2008. Cox regression analysis was performed to assess all-cause mortality associations. To evaluate the discriminatory power of the risk score, ROC curves were constructed for mortality at 1, 5, and 10 years for those with and without statin use.

**Results:**

103 patients were included, 69 of whom were taking statins. After 1, 5, and 10 years, survival rates predicted by the risk score for patients using statins were, respectively, 0.87 (95% CI [0.76;0.97]), 0. 45 (95% CI [0.37;0.55]), and 0.15 (95% CI [0.09;0.22]). Actual survival rates were 0.95, 0.88, and 0.72. For the 34 patients who did not use statins, predicted survival rates were 0.84 (95% CI [0.71;0.97]), 0.43 (IC 95% [0.32;0.55]), and 0.14 (95% CI [0.05;0.22]); actual survival rates were 0.83, 0.36, and 0.29.

**Conclusion:**

Patients receiving statins had greater survival rate after 5 and 10 years when compared to calculations by the risk score. The 34 patients who did not use statins had survival rates close to the predicted survival. Therefore, the risk score should be modified to include use of statins.

## Introduction

Atherosclerotic renovascular disease (ARVD) is not a rare condition and, depending of the severity of the hemodynamic impairment, can result in renovascular hypertension, ischemic nephropathy, and renal failure^1,2^. Renovascular hypertension occurs in approximately 5% of hypertensive patients and atherosclerosis is the most common cause of renal artery stenosis (RAS)^3^. In ARVD, a progressive involvement of the microcirculation with inflammation and fibrosis occurs, causing irreversible lesions in the renal parenchyma and development of chronic kidney disease (CKD)^4^.

Cardiovascular disease is the major cause of mortality in patients with ARVD due to associated coronary artery disease (CAD) and cerebrovascular disease, as atherosclerosis is a systemic disease^5^.

Renal revascularization is recommended as treatment only for a selected minority of patients^6^, based upon the severity of RAS, the state of the kidney beyond the RAS, and the clinical presentation of the patient^7^. For most patients, the mainstay of treatment involves renin-angiotensin-aldosterone system (RAAS) inhibition and statin use. Optimized medical therapy is associated with lower rates of CKD progression and lower mortality in patients with ARVD^8,9^.

Previously, Hagemann et al.^10^ investigated whether renal revascularization could improve renal outcomes in a Brazilian cohort of patients with RAS. Their results reinforced that only a carefully selected subset of patients, especially those with progressive worsening of renal function, could benefit from the procedure. At the same time, the study also confirmed the importance of medical therapy with angiotensin converting enzyme inhibitors (ACEi) or aldosterone receptors blockers (ARB) to inhibit renal function decline in patients with RAS.

Vassallo et al.^11^ developed a clinical risk calculator to predict the probability of major clinical outcomes in patients with ARVD. In that study, 872 patients with ARVD were followed up for an average period of 54.9 months and the evaluated outcomes were overall mortality, adverse cardiovascular events, and progression to end-stage kidney disease. A risk score was created based on the following variables: age, estimated glomerular filtration rate (eGFR), proteinuria, renal revascularization, history of myocardial infarction, left ventricular failure, and peripheral arterial disease. Statin use was not included in the model. The risk calculator is an important tool to help devise a patient-specific therapeutic approach, although it requires validation in different populations.

The aim of the present study was to evaluate the mortality rate of patients with ARVD in a Brazilian cohort and compare with the risk obtained from the risk calculator, but including the use or not of statins.

## Methods

The present analysis was a retrospective study of ARVD patients treated at a specialized hypertension center in Brazil and the primary outcome was all-cause. This cohort was previously analyzed by Hagemann et al.^10^. Patients were evaluated using the risk score calculator developed by Vassallo et al.^11^ to access the validity of the score.

Patients older than 18 years with RAS of the renal artery diameter above 60% unilaterally or bilaterally, as evidenced by direct catheter angiography, were included. Those with non-atherosclerotic RAS were excluded.

Patient data at baseline included age, race, gender, smoking status, diabetes mellitus (DM), CAD, peripheral arterial disease, chronic heart failure class as per New York Heart Association Classification, creatinine, estimated glomerular filtration rate (eGFR) by the CKD-EPI formula, proteinuria (24h urine protein excretion), renal revascularization, use of angiotensin converting enzyme inhibitor (ACEi) or angiotensin receptor blocker (ARB), use of statins and use of beta-blockers.

To calculate the risk score as per Vassallo et al.^11^, the following variables were used: age, eGFR, proteinuria, DM, whether or not the revascularization procedure was performed, history of myocardial infarction, left ventricular failure, and peripheral artery disease.

The study was approved by the Research Ethics Committee of the Faculdade de Medicina de Botucatu, Unesp, and the need for informed consent term was waived, because this was a retrospective study using anonymized data.

Categorical variables are expressed as absolute values and percentages and continuous variables are expressed as mean ± standard deviation. Cox proportional regression was performed for survival analysis. Survival probabilities predicted by the risk score for each patient were calculated with the risk score calculator, as well as the mean and 95% confidence intervals for one-, 5-, and 10-year survival. The predicted survival curves were compared to the actual survival curves. A p value < 0.05 was considered statistically significant. The statistical software used was SPSS 25.0.

## Results

From January 1996 to July 2008, 136 patients with angiographic diagnosis of ARVD were followed at the Special Hypertension Outpatient Clinic of the Faculdade de Medicina de Botucatu – Unesp. Of these patients, 103 were included and their baseline characteristics are shown in Table 1. The other 33 patients were not included in the study due to insufficient data or because they did not meet the inclusion criteria.

**Table 1:** Baseline characteristics of all patients

Age, years	65.6 ± 9.95
Creatinine, mg/dL	1.63 ± 1.24
Estimated glomerular filtration rate, mL/min/1.73 m^2^	39.26 ± 24.11
Proteinuria, g/24h	2.37 ± 1.90
LVF NHYA class	2; 3
Male n (%)	55 (53.4)
Black	10 (9.7)
Smoking	70 (68)
Diabetes	27 (26.2)
Dyslipidemia	90 (88.2)
Coronary artery disease	49 (47.6)
Peripheral artery disease Bilateral RAS Average % RAS right kidney Average % RAS left kidney	61 (59.2) 54 (52.4) 63.84 ± 37.1 67.33 ± 37.2
Renal revascularization Bilateral revascularization Medical treatment only	54 (52.4) 5 (4.8) 49 (47.5)
Use of ACEi/ARB	75 (72.8)
Use of statins	69 (67)
Use of betablocker	60 (58.25)

Abbreviations – ACEi: angiotensin converting enzyme inhibitor; ARB: angiotensin receptor blocker.

**Figure 1:**
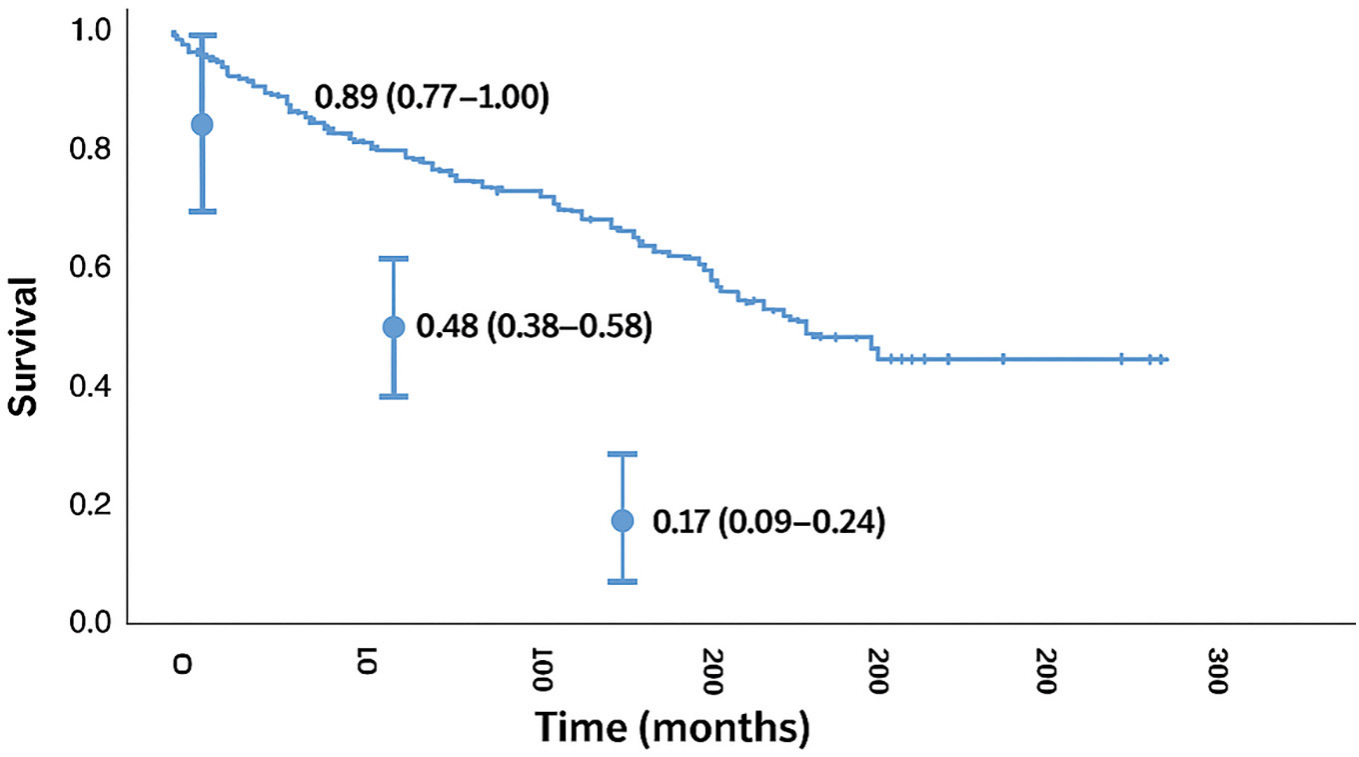
Cumulative survival curve^1^ compared with the expected curve^2^ according to the risk score developed by Vassallo et al. for 12, 60, and 120 months follow up.

**Figure 2:**
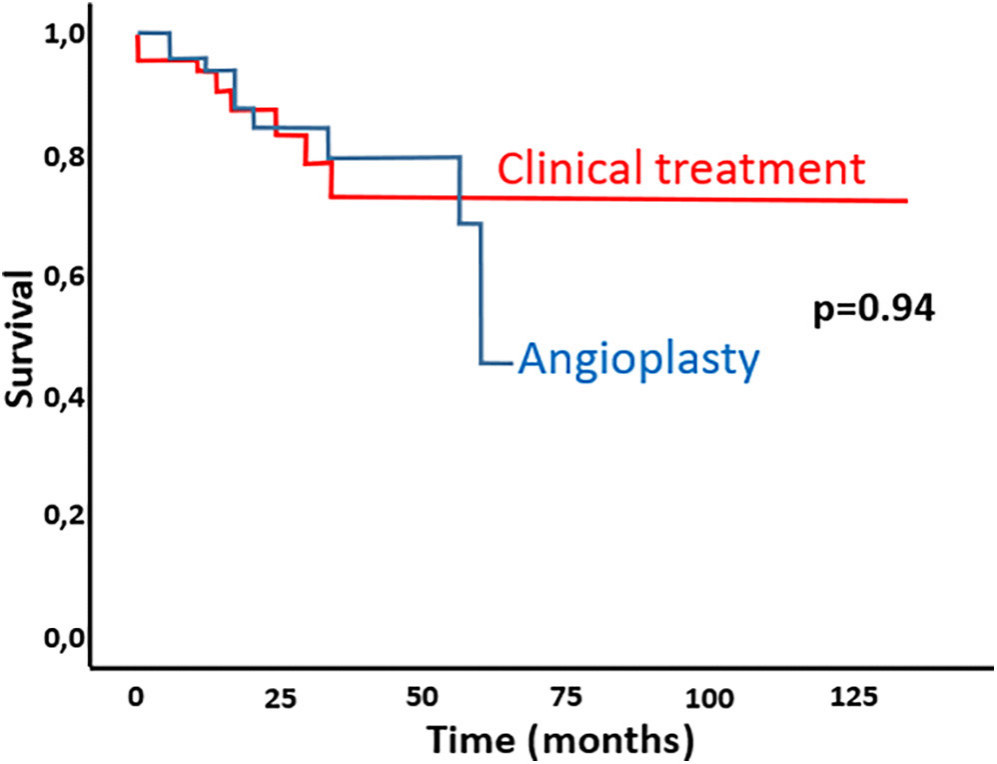
Cumulative survival curve comparing patients undergoing renal revascularization with patients not receiving revascularization.

The average age at baseline was 65.6 years and all-cause mortality was 50% at 150 months (Figure 1). Of the 103 patients, 54 were revascularized, while 49 received medical treatment only. According to Cox Regression survival curves, the observed survival at 12 months was similar to that predicted by the model. The predicted survival was 0.89 (95% CI [0.77;1.00]) while the actual survival rate was 0.92, therefore within the confidence interval. However, the observed mortality at 60 and 120 months was different from that predicted by the model: the predicted survival rates were, respectively, 0.48 (95% CI [0.38;0.58]) and 0.17 (95% CI [0.09;0.24) and the actual survival rates were 0.82 and 0.64, well above the confidence interval of the predicted values. There was no difference in the mortality rates, or time of follow-up, between the patients who underwent revascularization with angioplasty and stenting from those who only received medical management (Figure 2).

To investigate why our study cohort of 103 patients had higher probability of survival after five and 10 years than that predicted by the model, we undertook an analysis by use of statins: 69 patients were taking statins. The baseline characteristics of the groups of patients taking and not taking statins are shown in Table 2. Since the study is a historical cohort, some patients were not taking statins, as the use of this medication was not as well established in the past as it is today.

**Table 2:** Baseline characteristics of patients taking and not taking statins

	Statin	No statin	P
Age	65.3 ± 98	66.2 ± 10.3	0.47
Creatinine	1.9 ± 0.93	3.3 ± 2.8	<0.01
eGFR	42 ± 22.1	33.6 ± 27.1	0.12
Male	33 (48)	22 (65)	0.11
Black	9 (13)	4 (12)	0.83
Smoking	50 (72.5)	20 (59)	0.42
Diabetes	17 (24.6)	10 (29)	0.60
Dyslipidemia	67 (97)	23 (68)	<0.01
Coronary artery disease	38 (55)	11 (32)	0.03
Peripheral artery disease	45 (65)	16 (47)	0.08
Bilateral RAS	34 (49)	21 (62)	0.23
Renal revascularization	36 (52)	18 (53)	0.94
Bilateral revasculariation	2 (3)	3 (9)	0.19
Use of ACEi/ARB	55 (80)	20 (59)	0.03
Use of betablocker	47 (68)	13 (38)	<0.01
Death	4 (5.8)	13 (38)	<0.01

**Figure 3:**
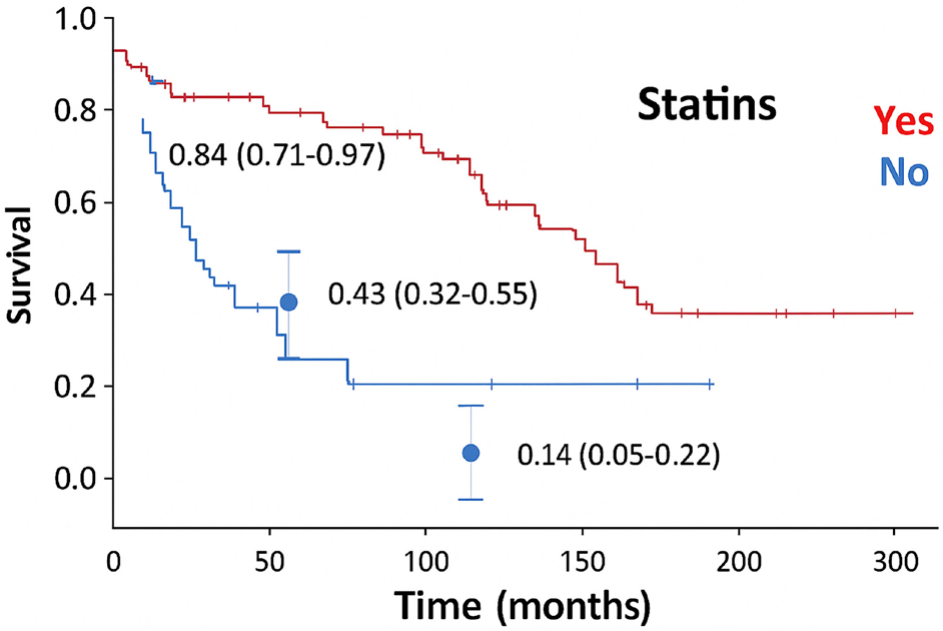
Cumulative survival curve of patients stratified by statin use.

A new Cox regression curve was performed (Figure 3) to compare outcomes between patients who used statins and those who did not. For the 34 patients who were not on statins, the survival curves generated by the regression model were very similar to those predicted by the model of Vassallo et al.^11^. Actual survival and predicted survival was 0.83 and 0.84 (95% CI [0.71;0.97]), 0.36 and 0.43 (95% CI [0.32;0.55]) and 0.29 and 0.14 (95% CI [0.05;0.22]) after 12, 60, and 120 months, respectively. Patients who were taking statins had substantially higher survival rates than the rate predicted by the risk score. The observed and predicted survival was 0.95 and 0.87 (95% CI [0.76;0.97]) after 12 months, 0.88 and 0.45 (95% CI [0.37;0.55]) after 60 months, and 0.72 and 0.15 (95% CI [0.09;0.22]) after 120 months.

Among patients on statin therapy, a total of four deaths were recorded: three attributed to cardiovascular causes and one to an infectious cause. In contrast, among patients not on statins, thirteen deaths were reported. Of these, six were due to cardiovascular causes, three to infectious causes, three to undetermined causes, and one to neoplastic disease.

## Discussion

The present study evaluated whether the risk score developed by Vassallo, Foley and Kalra^11^ predicted survival rates of patients with ARVD in a Brazilian cohort from the Botucatu Nephrology Service. The findings indicated that the observed survival curve after one year of follow-up was similar to the predicted curve. After 5 and 10 years of follow-up, however, the observed survival rates were higher than those predicted by the risk calculator, and further analyses showed that this was largely associated with the use of statins.

One of the hypotheses for the difference observed in the long-term mortality is the improvement of clinical treatment over the years. Some participants from the Vassallo et al’s^11^ cohort initiated follow-up in the 1980s, when clinical treatment for atherosclerotic disease was not yet well established. Botucatu’s cohort initiated the follow-up in the second half of the 1990s, when the use of statins, beta-blockers, and RAAS inhibitors was widespread.

In patients not using statins, survival rates were close to those predicted by the risk calculator. While the present study involved the analysis of data collected between 1996 and 2008, Vassallo et al.’s^11^ risk score was developed based on a cohort with data between the years 1986 and 2014, without accounting for the use of statins or any medication history other than number of anti-hypertensive agents.

Other studies also demonstrated the impact of medical treatment in patients with ARVD, including the benefits of statins use in reducing mortality. Silva et al.^9^ performed a retrospective study to compare the overall survival and renal survival of 104 patients with ARVD, divided into a group that used statin (n = 68) and another one that did not use the drug (n = 36). The patients were followed-up for 11 years and their baseline characteristics, such as age and glomerular filtration rate, were similar to the patients included in the present study. Besides the lower mortality rates, patients using statins also had a lower rate of progression to CKD.

Hackam et al.^12^ also analyzed the impact of statin therapy on cardiovascular and renal events in a cohort of ARVD patients over 65 years. After 13 years of follow-up, the researchers identified that the use of the drug was associated with better prognosis with regards to myocardial infarction, acute renal failure, and end-stage kidney disease.

With regards to renin-angiotensin-aldosterone system (RAAS) blockers, Losito et al.^8^ reported higher survival rates after a five-year follow up in patients with ARVD using ACEi.

Dregoesc et al.^13^ followed 65 patients undergoing renal revascularization between 2004 and 2014 who had baseline characteristics similar to the population of present study regarding age in years (64.6 ± 8.3 vs. 65.6 ± 9.95 in the present study), male sex (47.6 vs. 53.4%), CAD prevalence (53.8 vs. 47.6%), and dyslipidemia (78.4 vs. 88.2%). After 120 months, survival rate was 65.4%, close to that of the present study (64%). In another study, Chrysochou et al.^14^ followed 82 patients with ARVD for 40.2 ± 16.6 months to evaluate the use of the N-terminal portion of the brain natriuretic peptide prohormone (Nt-proBNP) and troponin as predictors of cardiovascular events and mortality. Baseline characteristics were similar to our study, with age (72 ± 7 vs. 65.6 ± 9.95), prevalence of DM (22 vs. 26.2%), estimated GFR (38 ± 20 vs. 39.26 ± 24.11 mL/min/1.73 m^2^) and prevalence of CAD (61 vs. 47.6%). Mortality was 9% per year and at the end of follow-up the survival rate was 62.2%.

The present study has limitations, especially the small number of patients recruited from a single center. However, comprehensive access to patient data contributed to better accuracy of survival curves.

In conclusion, the present study found a mortality rate of 50% at 150 months in ARVD patients, significantly lower than that reported in the literature. The predictive score developed by Vassallo, Foley and Kalra underestimated the survival probability of the patients, indicating that it should not be used in this population. Patients who did not use statins had survival rates close to those predicted by the score, which suggests that use of statins should be included in a modified version of the risk calculator.

## Data Availability

The full set of anonymized data supporting the findings of this study has been made available on Mendeley Data and can be accessed via DOI [10.17632/d8pctby6hw.1].

## Supplementary Material

The following online material is available for this article:

Data used in the article are available and will be submitted as supplementary material.
