# Supplementary material for: Long-term mortality and predictive score performance in Brazilian atherosclerotic renovascular disease patients
Source: J Bras Nefrol. 2025 Oct 10;47(4):e20240181. doi: 10.1590/2175-8239-JBN-2024-0181en (PMC12517691; doi:10.1590/2175-8239-JBN-2024-0181en)
Supplement: Translation

# Translation: Mortalidade a longo prazo e desempenho de escores preditivos em pacientes brasileiros com doença renovascular aterosclerótica

**DOI:** 10.1590/2175-8239-JBN-2024-0181pt

**Authors:** Julia Gheller Salome, Lucas Peres Moraes, Rodrigo Hagemann, Vanessa dos Santos Silva, Fabio Cardoso Carvalho, Roberto Jorge da Silva Franco, Diana Vassallo, Luis Cuadrado Martin, Philip A. Kalra, Pasqual Barretti

**Affiliations:** 1 Universidade Estadual Paulista, Faculdade de Medicina de Botucatu, Departamento de Clínica Médica, Serviço de Nefrologia, Botucatu, SP, Brasil.; 2 Universidade Federal do Paraná, Departamento de Clínica Médica, Serviço de Nefrologia, Curitiba, SP, Brasil.; 3 Mater Dei Hospital, Department of Medicine, Msida, Malta.; 4 University of Manchester, Salford Royal Hospital, Salford, United Kingdom.

**Keywords:** Aterosclerose, Estenose da Artéria Renal, Doença Renal Crônica, Hipertensão, Mortalidade

## Abstract

**Introdução:**

Doença renovascular aterosclerótica (DRVA) pode causar estenose da artéria renal, hipertensão e doença renal crônica. Como o procedimento de revascularização para DRVA é controverso, desenvolveu-se um escore de risco para predizer mortalidade em pacientes acometidos, que requer validação em diferentes populações. O escore original não incluía uso de estatinas; portanto, o objetivo foi avaliar a precisão do escore de risco em pacientes com DRVA segundo a ingestão de estatinas.

**Métodos:**

Estudo longitudinal retrospectivo envolvendo 136 pacientes com diagnóstico angiográfico de EAR > 60% entre janeiro de 1996 e outubro de 2008. Realizou-se análise de regressão de Cox para avaliar associações com mortalidade por todas as causas. Para avaliar poder discriminatório do escore de risco, construíram-se curvas ROC para mortalidade em 1, 5 e 10 anos para aqueles com e sem uso de estatina.

**Resultados:**

Foram incluídos 103 pacientes; 69 faziam uso de estatinas. Após 1, 5 e 10 anos, taxas de sobrevida previstas pelo escore de risco para pacientes em uso de estatinas foram, respectivamente, 0,87 (IC95% [0,76;0,97]), 0,45 (IC95% [0,37;0,55]) e 0,15 (IC95% [0,09;0,22]). Taxas de sobrevida real foram 0,95, 0,88 e 0,72. Para os 34 pacientes que não utilizaram estatinas, taxas de sobrevida prevista foram 0,84 (IC95% [0,71;0,97]), 0,43 (IC95% [0,32;0,55]) e 0,14 (IC95% [0,05;0,22]); taxas de sobrevida real foram 0,83, 0,36 e 0,29.

**Conclusão:**

Pacientes recebendo estatinas apresentaram maior taxa de sobrevida após 5 e 10 anos comparados aos cálculos do escore de risco. Os 34 pacientes que não utilizaram estatinas apresentaram taxas de sobrevida próximas à sobrevida prevista. Portanto, o escore de risco deve ser modificado para incluir estatinas.

## Introdução

A doença renovascular aterosclerótica (DRVA) não é uma condição rara e, dependendo da gravidade do comprometimento hemodinâmico, pode resultar em hipertensão renovascular, nefropatia isquêmica e insuficiência renal^1,2^. A hipertensão renovascular ocorre em aproximadamente 5% dos pacientes hipertensos, sendo a aterosclerose a causa mais comum de estenose da artéria renal (EAR)^3^. Na DRVA, ocorre um envolvimento progressivo da microcirculação com inflamação e fibrose, causando lesões irreversíveis no parênquima renal e desenvolvimento de doença renal crônica (DRC)^4^.

As doenças cardiovasculares são a principal causa de mortalidade em pacientes com DRVA devido à doença arterial coronariana (DAC) e doença cerebrovascular associadas, uma vez que a aterosclerose representa uma doença sistêmica^5^.

A revascularização renal é recomendada como tratamento apenas para uma minoria selecionada de pacientes^6^, com base na gravidade da EAR, no estado do rim a jusante da EAR e na apresentação clínica do paciente^7^. Para a maioria dos pacientes, a base do tratamento envolve a inibição do sistema renina-angiotensina-aldosterona (SRAA) e o uso de estatinas. A terapia medicamentosa otimizada está associada a taxas mais baixas de progressão da DRC e menor mortalidade em pacientes com DRVA^8,9^.

Anteriormente, Hagemann et al.^10^ investigaram se a revascularização renal poderia melhorar os desfechos renais em uma coorte brasileira de pacientes com EAR. Seus resultados reforçaram que somente um subgrupo cuidadosamente selecionado de pacientes, especialmente aqueles com piora progressiva da função renal, poderia se beneficiar do procedimento. Ao mesmo tempo, o estudo também confirmou a importância da terapia medicamentosa com inibidores da enzima conversora da angiotensina (IECA) ou bloqueadores dos receptores de aldosterona (BRA) para inibir o declínio da função renal em pacientes com EAR.

Vassallo et al.^11^ desenvolveram uma calculadora de risco clínico para predizer a probabilidade de desfechos clínicos maiores em pacientes com DRVA. Nesse estudo, 872 pacientes com DRVA foram acompanhados por um período médio de 54,9 meses, e os desfechos avaliados foram mortalidade geral, eventos cardiovasculares adversos e progressão para doença renal em estágio terminal. Foi criado um escore de risco com base nas seguintes variáveis: idade, taxa de filtração glomerular estimada (TFGe), proteinúria, revascularização renal, história de infarto do miocárdio, insuficiência ventricular esquerda e doença arterial periférica. O uso de estatinas não foi incluído no modelo. A calculadora de risco constitui uma ferramenta importante para auxiliar na definição de uma abordagem terapêutica específica ao paciente, embora seja necessária sua validação em diferentes populações.

O objetivo do presente estudo foi avaliar a taxa de mortalidade de pacientes com DRVA em uma coorte brasileira e compará-la ao risco obtido pela calculadora de risco, porém incluindo o uso ou não de estatinas.

## Métodos

A presente análise foi um estudo retrospectivo de pacientes com DRVA atendidos em um centro especializado em hipertensão no Brasil, sendo o desfecho primário todas as causas. Essa coorte havia sido analisada anteriormente por Hagemann et al.^10^. Os pacientes foram avaliados por meio da calculadora de risco desenvolvida por Vassallo et al.^11^ para acessar a validade do escore.

Foram incluídos pacientes com idade superior a 18 anos com EAR superior a 60% do diâmetro, unilateral ou bilateral, comprovada por angiografia por cateter direto. Pacientes com EAR de origem não aterosclerótica foram excluídos.

Os dados basais dos pacientes incluíram idade, raça, sexo, tabagismo, diabetes mellitus (DM), DAC, doença arterial periférica, classe de insuficiência cardíaca crônica de acordo com a Classificação da *New York Heart Association*, creatinina, taxa de filtração glomerular estimada (TFGe) pela fórmula CKD-EPI, proteinúria (excreção urinária de proteína em 24 horas), revascularização renal, uso de inibidor da enzima conversora da angiotensina (IECA) ou bloqueador do receptor da angiotensina (BRA), uso de estatinas e uso de betabloqueadores.

Para o cálculo do escore de risco conforme Vassallo et al.^11^, foram utilizadas as seguintes variáveis: idade, TFGe, proteinúria, DM, realização ou não de revascularização, histórico de infarto do miocárdio, insuficiência ventricular esquerda e doença arterial periférica.

O estudo foi aprovado pelo Comitê de Ética em Pesquisa da Faculdade de Medicina de Botucatu, Unesp, e a necessidade de termo de consentimento livre e esclarecido foi dispensada, por se tratar de um estudo retrospectivo com uso de dados anonimizados.

As variáveis categóricas foram expressas em valores absolutos e porcentagens, e as variáveis contínuas foram expressas como média ± desvio padrão. A regressão proporcional de Cox foi realizada para a análise de sobrevida. As probabilidades de sobrevida previstas pelo escore de risco para cada paciente foram calculadas por meio da calculadora de escore de risco, bem como a média e os intervalos de confiança de 95% para 1, 5 e 10 anos de sobrevida. As curvas de sobrevida prevista foram comparadas às curvas de sobrevida real. Considerou-se estatisticamente significativo um valor de p < 0,05. O software estatístico utilizado foi o SPSS 25.0.

## Resultados

De janeiro de 1996 a julho de 2008, 136 pacientes com diagnóstico angiográfico de DRVA foram acompanhados no Ambulatório Especial de Hipertensão da Faculdade de Medicina de Botucatu – Unesp. Desses pacientes, 103 foram incluídos e suas características basais estão apresentadas na Tabela 1. Os demais 33 pacientes não foram incluídos no estudo devido à insuficiência de dados ou por não preencherem os critérios de inclusão.

**Tabela 1:** Características basais de todos os pacientes

Idade, anos	65,6 ± 9,95
Creatinina, mg/dL	1,63 ± 1,24
Taxa de filtração glomerular estimada, mL/min/1,73 m^2^	39,26 ± 24,11
Proteinúria, g/24h	2,37 ± 1,90
IVF – Classe NYHA	2; 3
Sexo masculino n (%)	55 (53,4)
Pretos	10 (9,7)
Tabagismo	70 (68)
Diabetes	27 (26,2)
Dislipidemia	90 (88,2)
Doença arterial coronariana	49 (47,6)
Doença arterial periférica EAR bilateral % média de EAR no rim direito % média de EAR no rim esquerdo	61 (59,2) 54 (52,4) 63,84 ± 37,1 67,33 ± 37,2
Revascularização renal Revascularização bilateral Tratamento exclusivamente medicamentoso	54 (52,4) 5 (4,8) 49 (47,5)
Uso de IECA/BRA	75 (72,8)
Uso de estatinas	69 (67)
Uso de betabloqueadores	60 (58,25)

Abreviaturas – IECA: inibidor da enzima conversora da angiotensina; BRA: bloqueador do receptor de angiotensina.

A média de idade basal foi de 65,6 anos e a mortalidade por todas as causas foi de 50% em 150 meses (Figura 1). Dos 103 pacientes, 54 foram submetidos à revascularização, enquanto 49 receberam apenas tratamento medicamentoso. De acordo com as curvas de sobrevida da Regressão de Cox, a sobrevida observada em 12 meses foi semelhante à prevista pelo modelo. A sobrevida prevista foi de 0,89 (IC95% [0,77;1,00]), enquanto a taxa de sobrevida real foi de 0,92, portanto dentro do intervalo de confiança. No entanto, a mortalidade observada aos 60 e 120 meses foi diferente da prevista pelo modelo: as taxas de sobrevida prevista foram, respectivamente, 0,48 (IC95% [0,38;0,58]) e 0,17 (IC95% [0,09;0,24) e as taxas de sobrevida real foram de 0,82 e 0,64, bem acima do intervalo de confiança dos valores previstos. Não houve diferença nas taxas de mortalidade ou no tempo de acompanhamento dos pacientes submetidos à revascularização com angioplastia e implante de *stent* em relação aos que receberam somente tratamento medicamentoso (Figura 2).

**Figura 1:** Curva de sobrevida cumulativa^1^ comparada à curva esperada^2^ de acordo com o escore de risco desenvolvido por Vassallo et al. para 12, 60 e 120 meses de acompanhamento.

**Figura 2:** Curva de sobrevida cumulativa comparando pacientes submetidos à revascularização renal com pacientes que não receberam revascularização.

Para investigar a razão pela qual nossa coorte de 103 pacientes apresentou maior probabilidade de sobrevida após cinco e dez anos em comparação à taxa prevista pelo modelo, realizamos uma análise com base no uso de estatinas: 69 pacientes faziam uso do medicamento. As características basais dos grupos de pacientes em uso e não uso de estatinas estão apresentadas na Tabela 2. Como se trata de um estudo de coorte histórica, alguns pacientes não faziam uso de estatinas, uma vez que a utilização desse medicamento não era tão comum no passado quanto é atualmente.

**Tabela 2:** Características basais dos pacientes em uso e não em uso de estatinas

	Estatina	Sem estatina	P
Idade	65,3 ± 98	66,2 ± 10,3	0,47
Creatinina	1,9 ± 0,93	3,3 ± 2,8	<0,01
TFGe	42 ± 22,1	33,6 ± 27,1	0,12
Sexo masculino	33 (48)	22 (65)	0,11
Pretos	9 (13)	4 (12)	0,83
Tabagismo	50 (72,5)	20 (59)	0,42
Diabetes	17 (24,6)	10 (29)	0,60
Dislipidemia	67 (97)	23 (68)	<0,01
Doença arterial coronariana	38 (55)	11 (32)	0,03
Doença arterial periférica	45 (65)	16 (47)	0,08
EAR bilateral	34 (49)	21 (62)	0,23
Revascularização renal	36 (52)	18 (53)	0,94
Revascularização bilateral	2 (3)	3 (9)	0,19
Uso de IECA/BRA	55 (80)	20 (59)	0,03
Uso de betabloqueador	47 (68)	13 (38)	<0,01
Óbito	4 (5,8)	13 (38)	<0,01

Uma nova curva de regressão de Cox foi realizada (Figura 3) para comparar os desfechos entre os pacientes que utilizaram estatinas e aqueles que não utilizaram. Para os 34 pacientes que não estavam em uso de estatinas, as curvas de sobrevida geradas pelo modelo de regressão foram muito semelhantes às previstas pelo modelo de Vassallo et al.^11^ A sobrevida real e a sobrevida prevista foram 0,83 e 0,84 (IC95% [0,71;0,97]), 0,36 e 0,43 (IC95% [0,32;0,55]) e 0,29 e 0,14 (IC95% [0,05;0,22]) após 12, 60 e 120 meses, respectivamente. Os pacientes que estavam sob tratamento com estatinas apresentaram taxas de sobrevida substancialmente mais elevadas do que a taxa prevista pelo escore de risco. As taxas de sobrevida observada e de sobrevida prevista foram de 0,95 e 0,87 (IC95% [0,76;0,97]) após 12 meses, 0,88 e 0,45 (IC95% [0,37;0,55]) após 60 meses e 0,72 e 0,15 (IC95% [0,09;0,22]) após 120 meses.

**Figura 3:** Curva de sobrevida cumulativa de pacientes estratificados pelo uso de estatina.

Entre os pacientes em terapia com estatinas, foram registrados quatro óbitos: três atribuídos a causas cardiovasculares e um a causa infecciosa. Em contraste, entre os pacientes que não estavam em uso de estatinas, foram relatados treze óbitos. Destes, seis foram decorrentes de causas cardiovasculares, três de causas infecciosas, três de causas indeterminadas e um por doença neoplásica.

## Discussão

O presente estudo avaliou se o escore de risco desenvolvido por Vassallo, Foley e Kalra^11^ era capaz de predizer as taxas de sobrevida de pacientes com DRVA em uma coorte brasileira do Serviço de Nefrologia de Botucatu. O s achados indicaram que a curva de sobrevida observada após um ano de acompanhamento foi semelhante à curva prevista. Após 5 e 10 anos de acompanhamento, no entanto, as taxas de sobrevida observadas foram superiores às previstas pela calculadora de risco, e análises adicionais demonstraram que esse resultado esteve amplamente associado ao uso de estatinas.

Uma das hipóteses para a diferença observada na mortalidade a longo prazo é a melhoria do tratamento clínico ao longo dos anos. Alguns participantes da coorte de Vassallo et al’s^11^ iniciaram o acompanhamento na década de 1980, quando o tratamento clínico para a doença aterosclerótica ainda não estava bem estabelecido. A coorte de Botucatu iniciou o acompanhamento na segunda metade da década de 1990, quando o uso de estatinas, betabloqueadores e inibidores do SRAA estava amplamente difundido.

Em pacientes que não utilizavam estatinas, as taxas de sobrevida foram próximas às previstas pela calculadora de risco. Embora o presente estudo tenha envolvido a análise de dados coletados entre 1996 e 2008, o escore de risco de Vassallo et al.’s^11^ foi desenvolvido com base em uma coorte cujos dados foram coletados entre 1986 e 2014, sem considerar o uso de estatinas ou qualquer histórico de medicação além do número de agentes anti-hipertensivos.

Outros estudos também demonstraram o impacto do tratamento medicamentoso em pacientes com DRVA, incluindo os benefícios do uso de estatinas na redução da mortalidade. Silva et al.^9^ realizaram um estudo retrospectivo para comparar a sobrevida global e a sobrevida renal de 104 pacientes com DRVA, divididos em um grupo que utilizou estatina (n = 68) e outro que não fez uso do medicamento (n = 36). Os pacientes foram acompanhados por 11 anos e suas características basais, como idade e taxa de filtração glomerular, foram semelhantes às dos pacientes incluídos no presente estudo. Além das taxas de mortalidade mais baixas, os pacientes que utilizaram estatinas também apresentaram menor taxa de progressão para DRC.

Hackam et al.^12^ também analisaram o impacto da terapia com estatinas em eventos cardiovasculares e renais em uma coorte de pacientes com DRVA com mais de 65 anos. Após 13 anos de acompanhamento, os pesquisadores identificaram que o uso do medicamento esteve associado a um melhor prognóstico em relação a infarto do miocárdio, insuficiência renal aguda e doença renal em estágio terminal.

No que se refere aos bloqueadores do sistema renina-angiotensina-aldosterona (SRAA), Losito et al.^8^ relataram taxas de sobrevida mais elevadas após um acompanhamento de cinco anos em pacientes com DRVA em uso de IECA.

Dregoesc et al.^13^ acompanharam 65 pacientes submetidos à revascularização renal entre 2004 e 2014, com características basais semelhantes às da população do presente estudo em relação à idade em anos (64,6 ± 8,3 vs. 65,6 ± 9,95 no presente estudo), sexo masculino (47,6 vs. 53,4%), prevalência de DAC (53,8 vs. 47,6%) e dislipidemia (78,4 vs. 88,2%). Após 120 meses, a taxa de sobrevida foi de 65,4%, valor próximo ao do presente estudo (64%). Em outra investigação, Chrysochou et al.^14^ acompanharam 82 pacientes com DRVA por 40,2 ± 16,6 meses para avaliar o uso da porção N-terminal do pró-hormônio do peptídeo natriurético cerebral (Nt-proBNP) e da troponina como preditores de eventos cardiovasculares e mortalidade. As características basais foram semelhantes às do nosso estudo, incluindo idade (72 ± 7 vs. 65,6 ± 9,95), prevalência de DM (22% vs. 26,2%), TFG estimada (38 ± 20 vs. 39,26 ± 24,11 mL/min/1,73 m^2^) e prevalência de DAC (61% vs. 47,6%). A mortalidade foi de 9% ao ano e, ao final do acompanhamento, a taxa de sobrevida foi de 62,2%.

O presente estudo apresenta limitações, especialmente o número reduzido de pacientes recrutados em um único centro. No entanto, o acesso abrangente aos dados dos pacientes contribuiu para uma maior precisão das curvas de sobrevida.

Em conclusão, o presente estudo constatou uma taxa de mortalidade de 50% em 150 meses em pacientes com DRVA - significativamente inferior à relatada na literatura. O escore preditivo desenvolvido por Vassallo, Foley e Kalra subestimou a probabilidade de sobrevida dos pacientes, indicando que ele não deve ser aplicado nesta população. Os pacientes que não utilizaram estatinas apresentaram taxas de sobrevida próximas às previstas pelo escore, o que sugere que o uso de estatinas deve ser incluído em uma versão modificada da calculadora de risco.

## Data Availability

O conjunto completo de dados anonimizados que suporta os achados deste estudo foi disponibilizado no *Mendeley Data* e pode ser acessado por meio do DOI \[10.17632/d8pctby6hw.1].

## Material Suplementar

O seguinte material online está disponível para este artigo:

Os dados utilizados no artigo estão disponíveis e serão submetidos como material suplementar.
